# Matrine inhibits BCR/ABL mediated ERK/MAPK pathway in human leukemia cells

**DOI:** 10.18632/oncotarget.22353

**Published:** 2017-11-10

**Authors:** Lingdi Ma, Zhenyu Xu, Jian Wang, Zhichao Zhu, Guibin Lin, Lijia Jiang, Xuzhang Lu, Chang Zou

**Affiliations:** ^1^ Laboratory Center, The Third People’s Hospital of Huizhou, Affiliated Hospital of Guangzhou Medical University, Huizhou 516002, China; ^2^ Department of Pharmacy, Yijishan Affiliated Hospital of Wannan Medical College, Wuhu 241001, China; ^3^ Laboratory Center, The Second People’s Hospital of Changzhou, Affiliated Hospital of Nanjing Medical University, Changzhou 213000, China; ^4^ Department of Hematology, The Second People’s Hospital of Changzhou, Affiliated Hospital of Nanjing Medical University, Changzhou 213000, China; ^5^ Clinical Medical Research Center, The Second Clinical College of Jinan University, Shenzhen People’s Hospital, Shenzhen 518020, China

**Keywords:** matrine, ERK/MAPK, BCR/ABL, chronic myelogenous leukemia (CML), K562 cells

## Abstract

The BCR/ABL fusion gene and its downstream signaling pathways such as Ras/Raf/MAPK, JAK/STAT3, and PI3K/AKT pathways play important roles in malignant transformation of leukemia, especially chronic myelogenous leukemia (CML). Our previous study showed that matrine, an alkaloid extracted from a Chinese herb radix sophorae, significantly inhibited the proliferation of human CML K562cells, induced cell cycle arrest in G0/G1, and promoted cell apoptosis. In the present study, we investigated the molecular mechanism of matrine in the growth inhibition of leukemia cells using K562 and HL-60 cell lines. RT-PCR and Western blot assay demonstrated that the expression of BCR/ABL in K562 and HL-60 cells was significantly inhibited by matrine treatment. Phosphorylation of MEK1, ERK1/2, and their upstream adaptor molecules Shc and SHP2 were significantly downregulated. The protein and mRNA expression of components of the ERK/MAPK signal pathway, and Bcl-xL, Cyclin D1, and c-Myc, were dramatically reduced. Conversely, the expression of p27, a negative regulator of cell cycle progression, increased after matrine treatment. These results indicated that the inhibition of ERK/MAPK and BCR/ABL signaling pathway was associated with matrine’s suppressive effects on the growth of K562 and HL-60 cells. In *in vivo* study, matrine significantly decreased the mortality rate of tumor-baring mice and suggested that matrine could exert its anti-leukemia effect *in vivo.*

## INTRODUCTION

Chronic myeloid leukemia (CML) is a hematopoietic stem cell malignancy characterized by a translocation between chromosomes 9 and 22 in which the translocated chromosome 22 is also known as the Philadelphia chromosome [1]. BCR/ABL is required for the pathogenesis of CML by activating multiple intracellular signal transduction pathways such as Ras/Raf/MAPK, JAK/STAT3, and PI3K/AKT via its protein tyrosine kinase (PTK) interfering with the development of hematopoietic stem cells or progenitor cells. Thus, targeting the signaling pathways activated by BCR/ABL is a promising approach for drug development to treat CML [2-5]. Imatinib mesylate (IM), a small molecule inhibitor of the BCR/ABL kinase, is very effective in the treatment of CML, but the high risks of drug toxicity, drug resistance, and recurrence after drug withdrawal greatly limit its application in accelerated and blastic CML [6, 7]. A better understanding of molecular mechanisms underlying the human hematopoietic progenitor transformation in CML is essential to developing alternative approaches to target leukemogenic cells in CML.

Among the downstream signaling pathways activated by BCR/ABL tyrosine kinase, the abnormal activation of mitogen-activated protein kinase (MAPK) pathway plays an important role in the occurrence and development of CML [8, 9]. MAPK is a member of a protein kinase family with highly conservative serine/threonine protein kinase, which could be activated by a series of extracellular signaling molecules and stimulus such as growth factors and cytokines as well as osmotic pressure changes. MAPK is involved in the regulation of cell proliferation and differentiation. There are three branches of the MAPK cascade, including extracellular signal regulated kinase (ERK), c-Jun N-terminal kinase (JNK), and p38 MAP kinase, that are activated by different up-stream signals and exert independent effects on regulations of distinct physiological processes. These three branches can be activated by various cell stimuli, such as growth factors, cytokines, and cellular stress. ERK mainly responds to growth factors and promotes cell growth. JNK and p38 MAPK have multiple effects, depending on the stimulus and the environment in which activations occur. Among these pathways, ERK/MAPK pathway is considered to be a classic MAPK signaling cascade. The general scheme is as follows: epidermal growth factor (EGF) in plasma binds to its specific receptor on the cell membrane, leading to its dimerization as it binds to the Src homology-2 domain of the adapter protein, growth factor receptor bound protein2 (Grb2), on the cell membrane by self-phosphorylation. With the formation of Grb2-SOS complex by Grb2 and G protein exchange factor (SOS), the Ras-GDP protein is recruited to the cell membrane and promotes the dissociation of Ras from GDP, which then binds to GTP to induce the formation of an active Ras-GTP complex. The activated Ras sequentially activates the downstream serine/threonine protein kinase including Raf-1, MEK (a MAP kinase), and MAPKs (ERK, JNK and p38MAPK). It is involved in the regulation of cell survival, cell proliferation, cell differentiation, cell cycle progression, and induction of leukemogenesis [10-13].

Matrine is an alkaloid extracted from Chinese herb radix sophorae. It has potent anti-inflammatory, anti-oxidative, anti-infective and immune regulatory effects [14-16]. Studies revealed that matrine might exert inhibitory effects on the growth of many kinds of tumors such as gastric cancer, rhabdomyosarcoma, acute myeloid leukemia, and breast cancer [17, 18]. In a previous study, we found that matrine effectively inhibited the proliferation of CML K562 cells and induced cell apoptosis. Further study indicated that matrine might inhibit the proliferation of leukemia cells via suppressing intracellular DNA synthesis, blocking mitosis, and inducing apoptosis [19]. Recent studies showed that matrine was able to inhibit the Ras/MAPK signaling pathway to exert anti-tumor effect on gastric cancer [20]. Although the anti-tumor effect of matrine has been studied in recent years, the mechanism underlying this effect is still poorly understood.

Here we investigated the potential molecular mechanism underlying the effects of matrine on CML K562 and HL-60 cells. Our results identified that anti-leukemic effects of matrine were correlated with the inhibition of ERK/MAPK signaling activation and decreased expression of upstream BCR/ABL, which suggested that matrine might be of therapeutic utility in patients with CML.

## RESULTS

### Matrine inhibited Ras/Raf/ERK/MAPK signaling through down-regulating phosphorylation of MEK1 and ERK1/2

**Figure 1:**
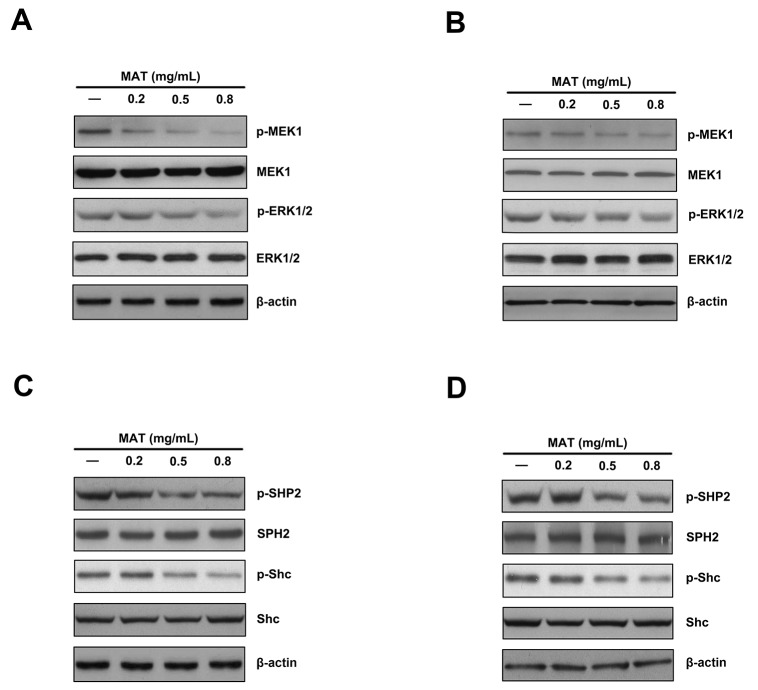
Matrine down-regulated the phosphorylation levels of effectors of ERK/MAPK signaling pathway in both K562 and HL-60 cells Matrine inhibited the expressions of phosphorylated MEK1 and ERK1/2. After 0.2, 0.5 and 0.8 mg/ml matrine treatment for 48h, the expressions of phospho-MEK and phospho-ERK1/2 were reduced significantly in K562 **(A)** and HL-60 cells **(B)**; **(C)** & **(D)**: Matrine down-regulated the expressions of phospho-SHP2 and phospho-Shc (two up-stream adaptor molecules of ERK/MAPK pathway). After matrine treatment for 48h, the protein expressions of phospho-SHP2 and phospho-Shc were reduced significantly.

Ras is an initial activator in Ras/Raf/ERK/MAPK signaling transduction pathway. Grb2 binds with SOS to become the Grb2-SOS complex, which activates Ras, and is then followed by activations of a MAP kinase kinase kinase (MKKK), activations of MAP kinase kinase (MKK) by MKKK, and finally activations of MAPK by MKK with phosphorylation [12, 13]. The protein of Src homology 2 domain (SPH2) and Src homology 2 domain containing protein C (Shc) play important roles in Grb2 recruitment and Ras activation [21, 22]. To assess whether the effect of matrine on leukemia cells is involved in the inactivation of Ras/Raf/ERK/MAPK signaling pathway, the expression of MEK, ERK1/2, phospho-MEK, phospho-ERK1/2 protein and the upstream adaptor molecules SHP2 and Shc were detected by Western blot. The ratio of phosphorylated protein to total protein was used to assess the influence of matrine on the protein phosphorylation. Our results showed that matrine treatment for 48h could significantly reduce the expressions of phosphorylated MEK1, ERK1/2 in both K562 and HL-60 cells without changing the protein expressions of total MEK1, ERK1/2 (Figure 1A and 1B).

In the classic Ras/Raf/MEK/ERK signaling pathway, Shc is an upstream molecule in the activation of Ras/MAPK pathway. The phosphorylation of tyrosine residues of Shc may provide a binding site to Grb2 to become a Shc-Grb2 complex. The cytoplasmic Grb2-SOS complex is recruited to the cell membrane, which leads to the activation of Ras-mediated MAPK cascade [22]. SHP2 is a non-receptor protein tyrosine phosphatase containing two Src homology-2 domains. Almost all cytokines- or growth factors-mediated Ras/MAPK activation is dependent on the activation of SHP2. EGFR, Grb2, and SOS may serve as substrates of SHP2 and directly bind to SHP2, leading to Ras activation. In addition, SHP2 may block the hydrolysis of Ras-GDP by GTP, which could prolong the half-life of Ras-GTP, resulting in the continuous activation of Ras/MAPK signaling pathway [21, 23]. It was also found in this study that matrine significantly reduced the expressions of phosphorylated Shc and SHP2 but failed to affect the expressions of total proteins of these molecules (Figure 1C and 1D). These results suggest that matrine might affect the cell growth and proliferation through regulating the activities of ERK/MAPK signaling pathway in both K562 and HL-60cells.

### Matrine down-regulated Cyclin D1, c-Myc, and Bcl-xL and, up-regulated p27

**Figure 2:**
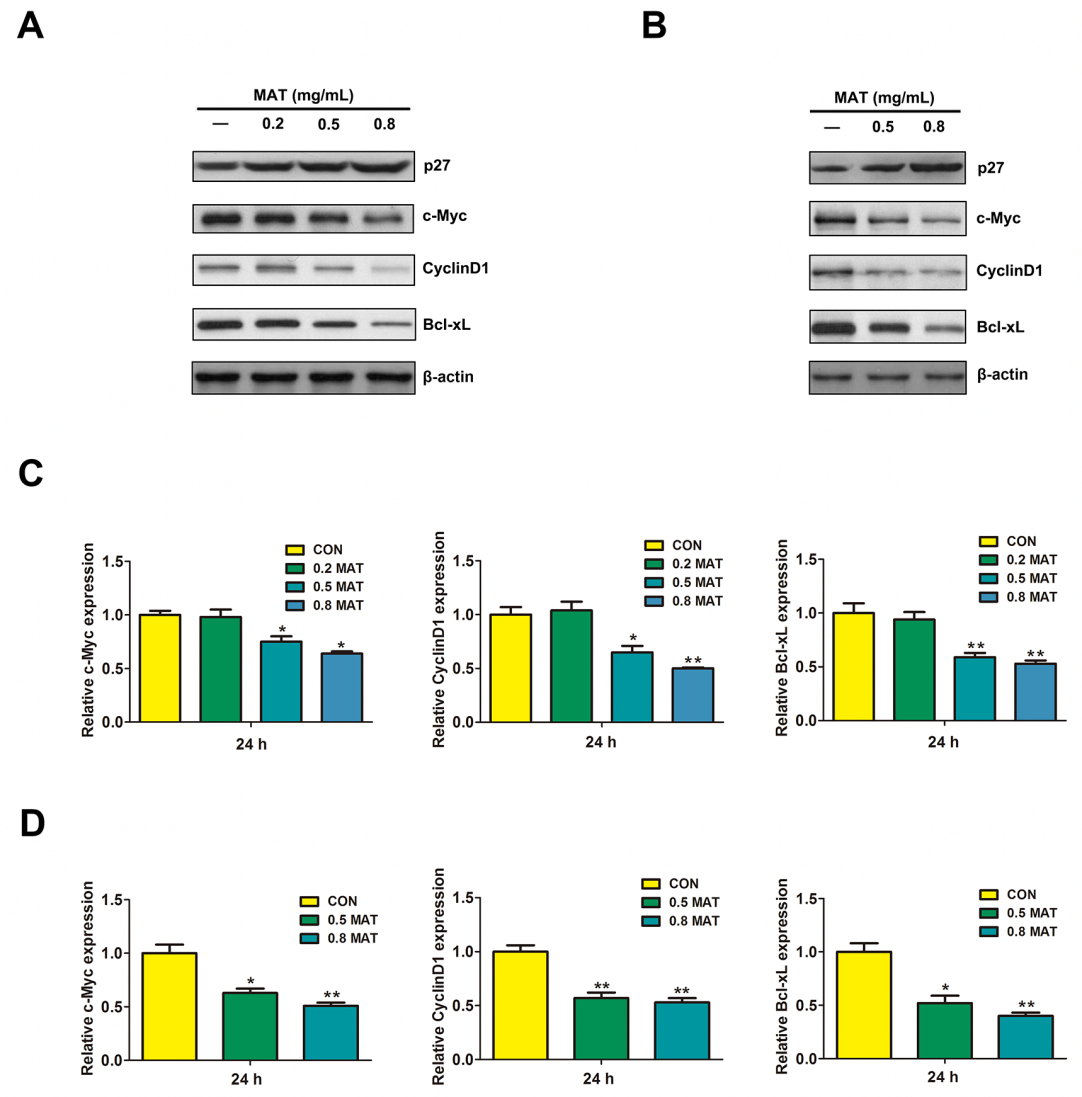
Effects of matrine on the expressions of molecules regulated by ERK/MAPK pathway Results of Western blot assay showed that 48h treatment with 0.5 and 0.8 mg/ml matrine significantly inhibited the expressions of c-Myc, Cyclin D1, and Bcl-xL proteins, but significantly increased p27 protein expression in K562 **(A)** and HL-60 cells **(B)**. RT-PCR results showed that 48h matrine treatment inhibited the mRNA expressions of c-Myc, Cyclin D1, and Bcl-xL in a dose-dependent manner in K562 **(C)** and HL-60 cells **(D)**. ^**^ p<0.01 vs. CON; ^*^ p<0.05 vs. CON.

We investigated the influence of matrine on Cyclin D1, c-Myc, p27, and Bcl-xL as they are important molecular factors involved in cell cycle progression, apoptosis, and proliferation. The Western blot results showed that matrine significantly reduced the expressions of cell cycle regulators Cyclin D1, c-Myc, and anti-apoptotic protein Bcl-xL. Under the same conditions, the negative regulator of cell cycle p27 protein was up-regulated in both K562 and HL-60 cells (Figure 2A and 2B). RT-PCR results confirmed the significantly attenuated expression of Bcl-xL, Cyclin D1, and c-Myc mRNA in both K562 and HL-60 cells in response to matrine treatment (Figure 2C and 2D). These findings were consistent with the cell proliferation inhibition and the induction of cell cycle arrest by matrine treatment in our previous study. Cyclin D1 plays a crucial role in the regulation of cell cycle progression, which could promote cells to cross the G1/S checkpoint and promote the cell cycle progression [24]. c-Myc, a product of oncogene c-Myc, may regulate the expressions of Cyclin D, E, and A, promote the cell cycle transition from G1 phase to S phase as well as regulate cell proliferation and apoptosis [25]. p27 is a negative regulator of cell cycle progression, which inhibits the formation of Cyclin D/CDK2 complex and block cells in G1 phase [26]. The above findings indicated that cell cycle blockage and apoptosis in K562 cells induced by matrine were related to the regulation of Cyclin D1, c-Myc, p27, and Bcl-xL expressions. Since these molecular factors could be responsive to the Ras/MAPK pathway [27-29], and the above results indicated that matrine significantly reduced phosphorylation levels of ERK, MEK, SHP2 as well as Shc, it suggested that matrine inhibited CML cell growth through the MEK/MAPK pathway.

### BCR/ABL were involved in matrine-induced inhibition of ERK/MAPK pathway

**Figure 3:**
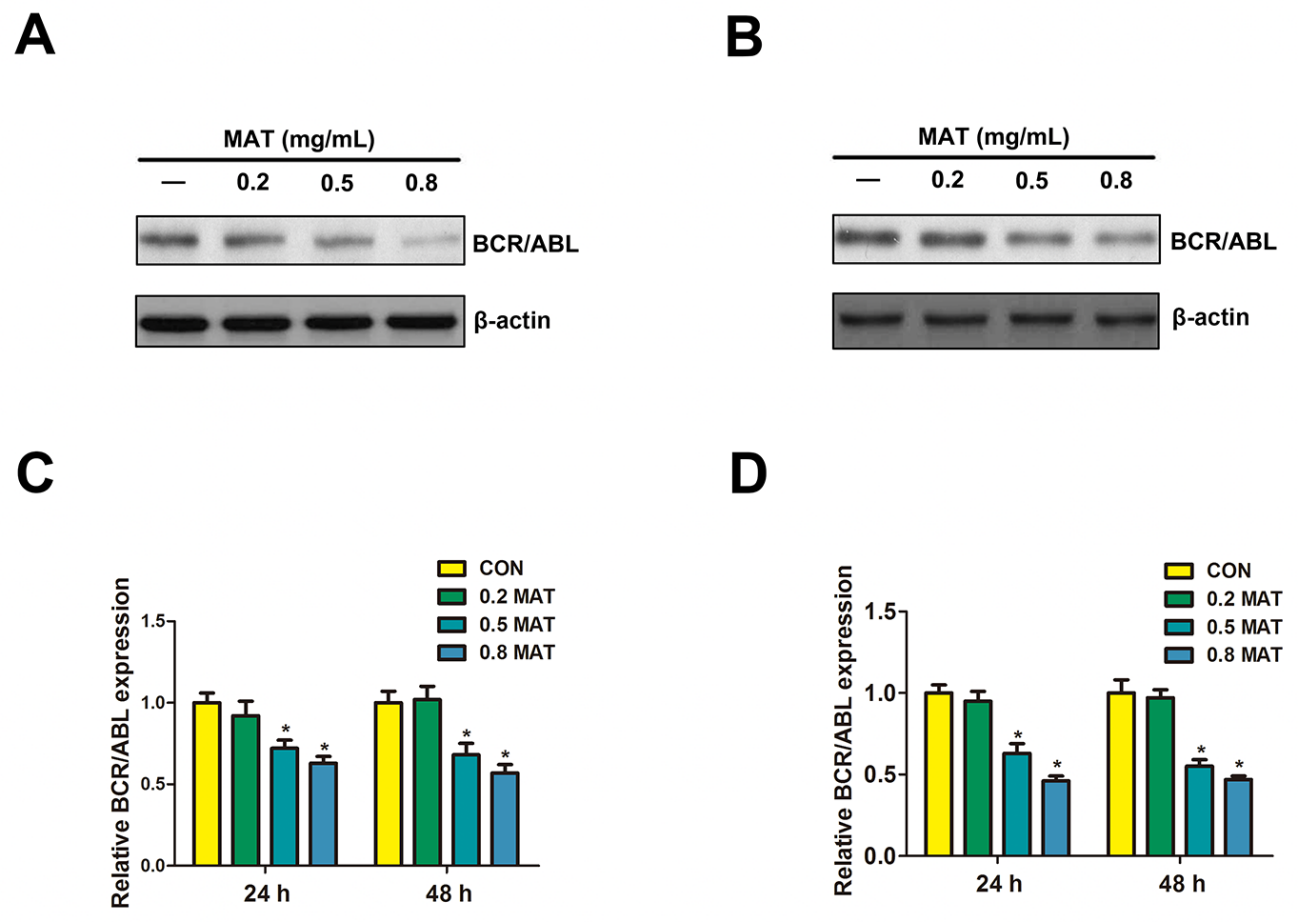
Matrine inhibited Bcr/Abl expression in K562 and HL-60 cells After matrine treatment for 48h, the protein in K562 **(A)** and HL-60 cells **(B)**, and mRNA expression in K562 **(C)** and HL-60 cells **(D)** levels of Bcr/Abl were reduced significantly when compared with the control group. ^**^ p<0.01 vs. CON; ^*^ p<0.05 vs. CON.

The oncogenic BCR/ABL tyrosine kinase is essential for leukemogenesis. BCR/ABL-mediated signal transduction pathways could interfere with various cellular physiological processes, including cells proliferation, transformation, and apoptosis, which made the BCR/ABL kinase an attractive target for clinical intervention in CML [4, 5]. There were extensive biochemical evidences suggesting that BCR/ABL activated ERK through the activation of the RAS/RAF/ERK/MAPK pathway [30, 31], but the mechanism by which matrine regulates the activation of ERK/MAPK pathway and whether this mechanism involves BCR/ABL are still unclear. To assess whether the effect of matrine on ERK/MAPK is involved in the activation of BCR/ABL-MAPK axes, Western blot was used to evaluate the expression of BCR/ABL fusion protein in both K562 and HL-60 cells. Results of this study demonstrated that matrine down-regulated BCR/ABL protein expression (Figure 3A and 3B) and mRNA levels of BCR/ABL (Figure 3C and 3D), indicating that anti-leukemic activities of matrine were related to BCR/ABL expression.

### Matrine down-regulated phosphorylated Src kinase

**Figure 4:**
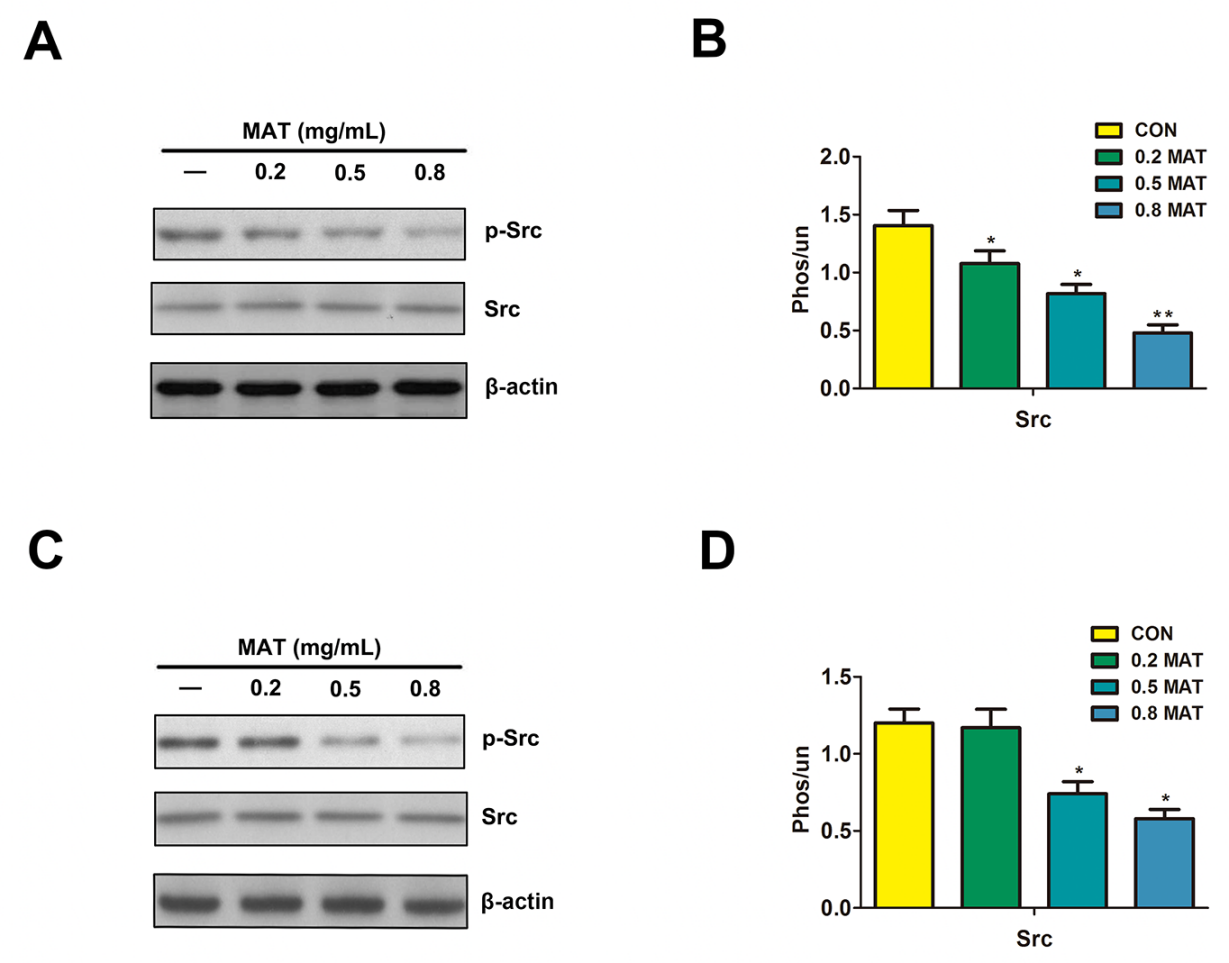
Matrine reduced the level of phosphorylated Src After treatment with matrine for 48h, the expression of phospho-Src level was reduced significantly inK562 **(A and B)** and HL-60 cells **(C and D)**. ^**^ p<0.01 vs. CON.

Src protein, a type of non-receptor protein tyrosine kinases, is a member of Src family kinases (SFK). Several reports indicated that SFKs played an important role in the pathogenesis of BCR/ABL+ myelogenous leukemia [32, 33]. To ascertain whether matrine plays a role in the modulation of Src activity, the expression of Src and phosphor-Src in both K562 and HL-60 cells after matrine treatment were tested. The results showed that treatment with matrine for 48h has no effects on the expression of total Src protein, but the expression of phosphor-Src was reduced significantly, suggesting that Src inhibition involved in the down regulating effects of matrine on the ERK/MAPK signaling pathway (Figure 4).

### Administration of matrine benefited total survival time of mice inoculated with human leukemia cells

**Figure 5:**
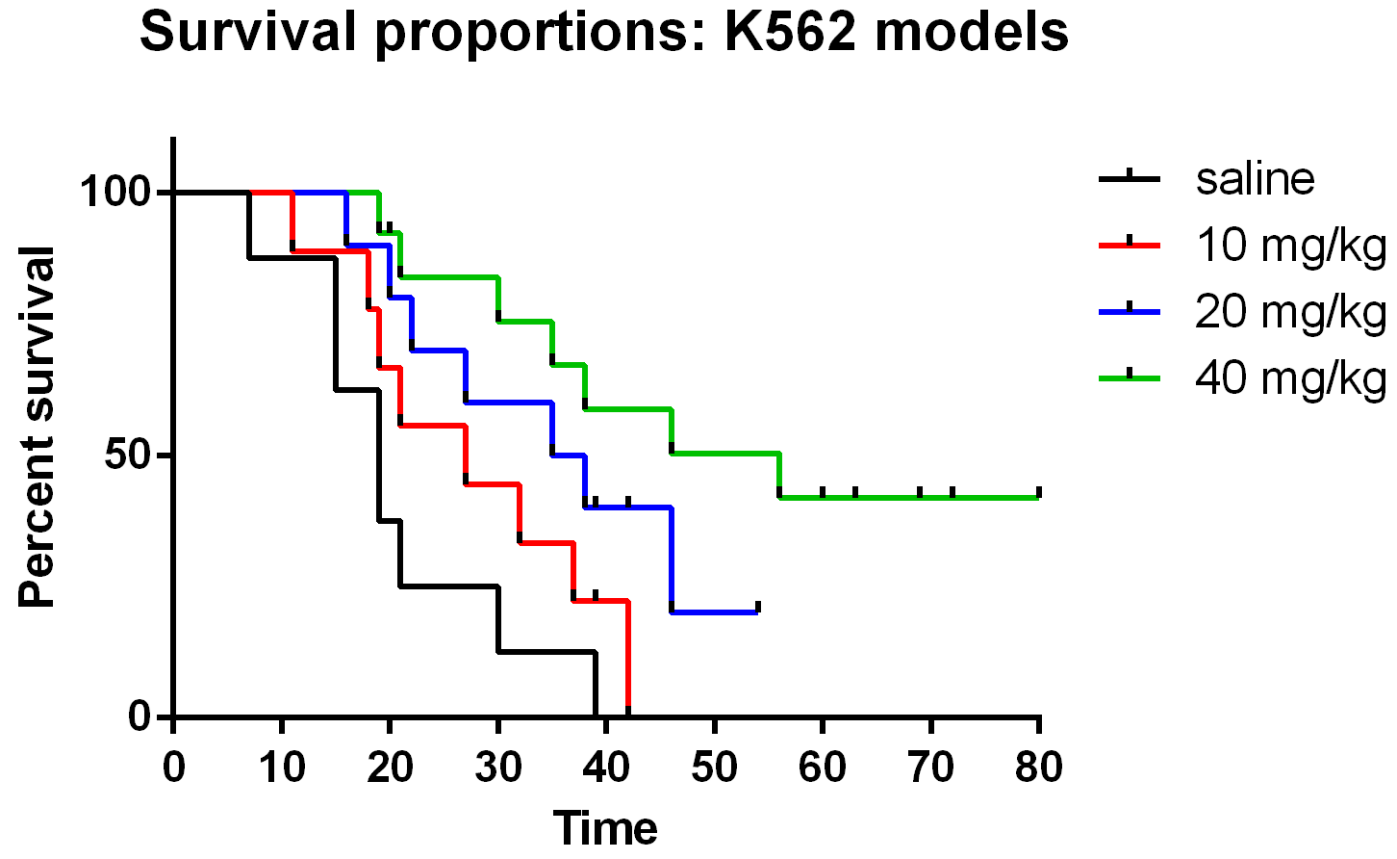
Matrine alleviates AML burden *in vivo* Administration of Matrine could benefit the total survival of mice inoculated with K562. Median survival of 10 mg/kg group, 20 mg/kg group and 40 mg/kg group is days,27 days and 36.5 days and 56 days separately, which are significant longer than the saline group (19 days, P<0.01).

Previously, we reported that matrine could inhibit the cell growth of human leukemia cells K562. To further confirm its anti-leukemia effect *in vivo*, matrine(10, 20, 40 mg/kg) as well as saline was injected in to mice with human leukemia cells very two days for 60 days. Survival rates were recorded and Kaplan–Meier survival curves showed that treatment of mice with matrine significantly decreased the mortality rate compared with mice that received saline in a dosage depend manner (Figure 5). These results indicated that matrine could exert its anti-leukemia effect *in vivo*.

## DISCUSSION

In a previous study, it was found that matrine induced a concentration-and time-dependent decrease in the number of human CML K562 cells with increasing cell apoptosis and significant G0/1→S cell cycle arrest, suggesting that matrine did not induce anti-proliferative effects through direct cytotoxicity [34], but through the blockage of cell cycle progression and induction of apoptosis. The aim of this study was to investigate whether matrine exerted its anti-leukemic effects through inhibiting the ERK/MAPK signaling pathway in both K562 and HL-60 cells. The cell cycle is mainly regulated in G1 phase, which determines the transition from G1 phase to S phase. Cyclin D1, c-Myc, and p27 are important proteins involved in the regulation of G1 phase. Analyses of the regulatory molecules revealed that matrine-induced G1 block was associated with a significant down-regulation of the cyclin D1 and c-Myc expression and increased expression of p27 (a cyclin-dependent kinase inhibitor). Since these molecules responds to the ERK/MAPK signaling pathway, the effects of matrine on ERK/MAPK pathway was then investigated. As expected, matrine treatment resulted in significantly reduced levels of phosphorylated MEK and ERK, the key signaling molecules of the Ras/Raf/ERK/MAPK oncogenic signaling pathway, thus confirming the potential suppressive effects of matrine on ERK/MAPK signaling. All these data suggested that anti-leukemic effects of matrine are mediated, at least in part, by inhibition of aberrantly activated ERK/MAPK signaling in both K562 and HL-60 cells.

The anti-apoptotic activity of BCR/ABL contributes greatly to the development of CML. BCR/ABL may function either by enhancing the proliferation potential of hematopoietic progenitors or by preventing these progenitor cells from apoptosis. These processes are thought to be involved in intracellular signaling pathways, such as ERK/MAPK, JAK/STAT3, and PI3K/AKT pathways. As a driving force for CML, the activated tyrosine kinase of BCR/ABL is able to stimulate the MAPK/ERK pathway. Studies have shown that after phosphorylation at Tyr177, BCR/ABL might bind to the Src homology-2 domain of Grb2 to create the BCR/ABL/Grb2 complex, which then recruit cytoplasmic SOS and switched into the BCR/ABL/Grb2/SOS complex, resulting in the activation of GDP-Ras [8, 35]. The activation of Ras resulted in activation of its downstream effectors such as Raf-1, MEK, and MAPK, and was responsible for the activation of ERK/MAPK pathway to promote myeloid and lymphoid transformation. Results showed that matrine treatment significantly inhibited the mRNA and protein expressions of BCR/ABL in dose- and time-dependent manners, suggesting that inhibition of BCR/ABL by matrine contributed to the growth inhibitions and the interruptions of ERK/MAPK signal network in both K562 and HL-60 cells.

Although BCR/ABL is considered the trigger of malignant transformation in CML, targeting BCR/ABL kinase activity alone may not be sufficient for the leukemogenesis of CML, for downstream pathways of BCR/ABL can be activated independently of BCR/ABL [36]. Reduction in phosphorylation levels could be the results of inhibition of other protein tyrosine kinases or activation of protein tyrosine phosphatase. The inhibitory effects of matrine on the phosphor tyrosine levels of ERK and MEK may not be precisely regulated by the suppression of upstream protein tyrosine kinases BCR/ABL. There was evidence of an important role of SFKs in CML progression. The SFKs intracellular non-receptor tyrosine kinases are collaborative oncogenic kinases in BCR/ABL-induced leukemia and may act to couple BCR/ABL to certain downstream signaling pathways involved in leukemic transformation. The activity of BCR/ABL can be enhanced through SFK-mediated phosphorylation within the activation loop of ABL, which was found to increase the ABL kinase activity in the progression of CML and Ph+ ALL. In BCR/ABL+CML cells, activated Src may induce the phosphorylation of BCR/ABL at Tyr89 and Tyr245 and further increase the tyrosine kinase activity of BCR/ABL [37]. Increasing preclinical and clinical evidence implicated that SFKs are also involved in imatinib resistance, which may be a cause of resistance to imatinib through BCR/ABL independently in Ph+ leukemia [38, 39]. SFKs are also related to the Ras-mediated activation of ERK/MAPK pathway. SHP2 could activate Src to phosphorylate up-stream adapter molecules, such as Shc, Grb2, and Gab2, leading to Ras/MAPK activation [40, 41]. In this study, we found that treatment with matrine had no influence on total Src protein expression, but reduced the phosphorylated Src significantly, suggesting that Src inhibition could be involved in the effects of matrineonbothK562 and HL-60 leukemic cells and that matrine may be a dual inhibitor for SFK and BCR/ABL.

In conclusion, we have documented that the underlying mechanisms of matrine’s anti-CML activity were involved in the inhibition of MAPK/ERK signaling pathway and down-regulated expression of BCR/ABL. Matrine is also found to inhibit the activation of Src kinase, suggesting that matrine may be an effective inhibitor of both SFK and MAPK/ERK. Further investigation of matrine on the MAPK/ERK-BCR/ABL-related signaling pathways would elucidate its molecular mechanisms underlying the anti-leukemic effects and its potential in the clinical therapy of CML and Ph+ ALL.

## MATERIALS AND METHODS

### Reagents and antibodies

Matrine with purity up to 99.5% was purchased from the Institute of Phytochemistry of Xi'an Botanical Garden in Shan’Xi Provincial Academy of Sciences. A stock solution (10mg/ml) was prepared by dissolving matrine in ddH2O and stored at −20°C.K562 and HL-60cells were obtained from Shanghai Cell Bank of Chinese Academy of Science,Shanghai, China, and maintained in RPMI l640 (Gibco, USA) containing 10% fetal bovine serum (Gibco, USA) in an environment with 5% CO2 at 37°C. Primers for BCR/ABL, Bcl-xL, CyclinD1, and c-Myc were designed by Shanghai Sangon Co., Ltd. Antibodies against BCR/ABL (3908s), Bcl-xL (2762), CyclinD (2922), c-Myc (9402), p27 (2552) were purchased from Cell Signaling Technology (Beverly, MA, USA). Monoclonal antibodies against MEK1(YT2711), phospho-MEK1 (Ser217)(YP0167), ERK1/2(YT1625), phospho-ERK1/2 (Tyr204)(YP0101), SHP2(YT4295), phosphor-SHP2(Tyr580)(YP0582), Shc(YT4287), phospho-Shc (Tyr349) (YP0578) were purchased from Immunoway Biotechnology (Immunoway, USA). Src (anti-4878) and phospho-Src (Tyr418) were from Abnova, Taiwan.

### Cell culture

K562 or HL-60 cells in logarithmic growth phase at 0.5-1.0×10^5^/ml were treated with matrine at 0.2 mg/ml, 0.5 mg/ml and 0.8 mg/ml. In the control group, PBS of an equal volume was added. Cells were maintained in a humidified environment with 5% CO2 at 37°C for 24 h and 48 h. Cells were collected, and total RNA and protein were extracted for RT-PCR and Western blot assay.

### Real-time reverse transcription-PCR

Real-time reverse transcription-PCR for estimation of BCR/ABL, Bcl-xL,CyclinD1, and c-Myc mRNAs were carried out by using the following primers (Table 1). Total RNA was extracted from matrine-treated and untreated K562 or HL-60 by using TRIzol reagent (Gibco, USA). Five micrograms of RNA was reversely transcribed into cDNA using Revert Aid™ First Strand cDNA Synthesis Kit (K1622; Fermentas Inc, USA). Transcribed cDNA was amplified and quantified by the real-time fluorescent quantitative PCR with a SYBR Green qPCR kit (TaKaRa). The relative expressions of every gene were assessed in comparison with the housekeeping gene glyceraldehyde-3-phosphate dehydrogenase (GAPDH). Blank controls, that did not contain cDNA, were run in parallel. All samples were run in triplicate. Relative expression values with SE and statistical comparison (unpaired two-tailed t test) were obtained by using the Qgene software.

**Table 1:** Sequence and size of primers for PCR

Gene	Sequences	Size (bp)
BCR/ABL	5’- CTCCAGACTGTCCACAGCATTCCG	168 or 93
	3’- CAGACCCTGAGGCTCAAAGTCAGA	
Bcl-xL	5’- CCCAGAAAGGATACAGCTGG	448
	3’- GCGATCCGACTCACCAATAC	
CyclinD1	5’- CTGGCCATGAACTACCTGGA	483
	3’- GTCACACTTGATCACTCTGG	
c-Myc	5’- CTCTCAACGACAGCAGCCCG	250
	3’- CCAGTCTCAGACCTAGTGGA	
GAPDH	5’- ACCACAGTCCATGCCATCAC	450
	3’- TCCACCACCCTGTTGCTGTA	

### Western blot

K562 or HL-60 cells were treated with matrine for 48 h, and cells were harvested and lysed by using RIPA lyses buffer (Beijing Biotech Co., Ltd) for the extraction of total protein. Protein concentration was quantified with BCA Protein Assay Kit (Merck Chemicals Shanghai Company). For the detection of target proteins and phosphorylated proteins, proper proteins were subjected to 12.5% SDS-PAGE at a constant of 30 mA and then transferred onto PVDF membrane at 4°C overnight. The membrane was blocked in 5% non-fat milk and then probed with various primary antibodies (1:500 or 1:2000) at 4°C overnight. β-actin was used as an internal reference. The membrane was washed with TBST three times and then incubated with HRP-conjugated secondary antibody (1:5000) at 37°C for 4h. Visualization was performed using the enhanced chemiluminescence detection reagents (Amersham Pharmacia Biotech). Film was exposed under X-ray. Representative images were captured.

### *In vivo* study

K562 cells (1^*^10^7^/mouse) were injected once via tail vein of NoD/SCID mice at 5-6 weeks old. 20 days after the tail vein injection, mice were divided into 4 groups randomly. Different dosages of matrine as well as saline were injected into each group by *i.p.* injection every two days accordingly. Total survival dates of each group were documented for two months and mice were sacrificed when they show dying signs.

### Statistical analysis

Data are presented as mean ± standard deviation $\left(\bar{x}\pm \mathrm{SD}\right)$. One-way ANOVA was used for comparisons among groups and q test with Newman-keuls method for paired comparisons. P value less than 0.05 was considered statistically significant and was derived from 2-tailed statistical test. All statistical analyses were performed using the software SPSS 14.0.
